# Impact of short-term traffic-related air pollution on the metabolome – Results from two metabolome-wide experimental studies

**DOI:** 10.1016/j.envint.2018.11.034

**Published:** 2019-02

**Authors:** Karin van Veldhoven, Agneta Kiss, Pekka Keski-Rahkonen, Nivonirina Robinot, Augustin Scalbert, Paul Cullinan, Kian Fan Chung, Peter Collins, Rudy Sinharay, Benjamin M. Barratt, Mark Nieuwenhuijsen, Albert Ambros Rodoreda, Glòria Carrasco-Turigas, Jelle Vlaanderen, Roel Vermeulen, Lützen Portengen, Soterios A. Kyrtopoulos, Erica Ponzi, Marc Chadeau-Hyam, Paolo Vineis

**Affiliations:** a MRC/PHE Centre for Environment and Health, Department of Epidemiology and Biostatistics, School of Public Health, Imperial College London, London, United Kingdom; b International Agency for Research on Cancer (IARC), Lyon, France; c National Heart & Lung Institute, Imperial College London, United Kingdom; d Royal Brompton & Harefield NHS Trust, London, United Kingdom; e King's College London, United Kingdom; f Barcelona Institute for Global Health (ISGlobal), Barcelona, Spain; g Institute for Risk Assessment Sciences (IRAS), Utrecht University, Utrecht, the Netherlands; h National Hellenic Research Foundation, Athens, Greece; i Epidemiology, Biostatistics and Prevention Institute, University of Zurich, Switzerland; j Italian Institute for Genomic Medicine (IIGM), Turin, Italy

**Keywords:** Traffic related air pollution, Metabolomics, Randomized crossover trials

## Abstract

Exposure to traffic-related air pollution (TRAP) has been associated with adverse health outcomes but underlying biological mechanisms remain poorly understood. Two randomized crossover trials were used here, the Oxford Street II (London) and the TAPAS II (Barcelona) studies, where volunteers were allocated to high or low air pollution exposures. The two locations represent different exposure scenarios, with Oxford Street characterized by diesel vehicles and Barcelona by normal mixed urban traffic. Levels of five and four pollutants were measured, respectively, using personal exposure monitoring devices. Serum samples were used for metabolomic profiling. The association between TRAP and levels of each metabolic feature was assessed. All pollutant levels were significantly higher at the high pollution sites. 29 and 77 metabolic features were associated with at least one pollutant in the Oxford Street II and TAPAS II studies, respectively, which related to 17 and 30 metabolic compounds. Little overlap was observed across pollutants for metabolic features, suggesting that different pollutants may affect levels of different metabolic features. After observing the annotated compounds, the main pathway suggested in Oxford Street II in association with NO_2_ was the acyl-carnitine pathway, previously found to be associated with cardio-respiratory disease. No overlap was found between the metabolic features identified in the two studies.

## Highlights

• Two randomized crossover trials were used to assess the relationship between TRAP and metabolic features with MS-based metabolomics (MWAS)
• The locations represent different exposure scenarios, with London characterized by diesel vehicles and Barcelona by normal mixed urban traffic
• Levels of 17 and 30 metabolic compounds associated with different air pollutants in the studies, with little overlap in features across pollutants
• No overlap found between metabolomic features identified in the two studies, possibly due to different levels of single pollutants
• The acyl-carnitine pathway, involved in cardio-respiratory disease, was suggested as a potential pathway in association with NO_2_ in one study

## 1 Introduction

Exposure to traffic-related air pollution (TRAP) has been associated with adverse respiratory and cardiovascular outcomes, both in healthy and susceptible subjects (W.H.O, 2013). Our previous observations in the experimental Oxford Street I study described a reduction of up to 6.1% in the forced expiration volume in 1 s (FEV_1_) and up to 5.4% in the forced vital capacity (FVC) among subjects walking in Oxford Street, a street in London with heavy exposure to diesel-related TRAP. These reductions were significantly larger compared with exposure in Hyde Park, a green area close to Oxford Street and subject to lower TRAP exposures (*p*-value = 0.04 and *p*-value = 0.01 respectively) (McCreanor et al., 2007). In a second independent study with the same design we concluded that the positive health effects of walking were inhibited in subjects with chronic obstructive pulmonary disease (COPD) or ischaemic heart disease (IHD) – and to a lower extent in subjects free of cardiopulmonary diseases - after short-term exposure to TRAP (Sinharay et al., 2017). Health effects of long-term exposure to TRAP have been studied extensively and the International Agency for Research on Cancer (IARC) reported in 2015 that there is “sufficient” evidence that ambient air pollution is carcinogenic to humans, and that the evidence is also “sufficient” for airborne particulates (International Agency for Research on Cancer (IARC) Outdoor Air Pollution, 2015). In addition, cardiovascular and pulmonary mortality have been associated with long-term exposure to air pollution (Hoek et al., 2013).

The mechanisms by which air pollution causes adverse health effects remain poorly understood. Inflammatory pathways or oxidative stress have been suggested to play a role but evidence is still sparse (Manney et al., 2012; Wu et al., 2014). Moller et al. noted that oxidatively damaged DNA is generated by particulate matter present in air pollution through the promotion of oxidative stress and inflammation (Møller et al., 2014) and Gawda et al. also concluded that PM-induced oxidative stress led to increased levels of inflammation (Gawda et al., 2017). The Oxford Street I study reported that biomarkers of neutrophilic inflammation and airway acidification were higher after exposure in Oxford Street than in Hyde Park (*p*-value = 0.05 and *p*-value = 0.003 respectively) (McCreanor et al., 2007). Airway inflammation was also observed among healthy subjects, as well as systemic effects such as an increase in neutrophil granulocytes (Jacobs et al., 2010; Riediker et al., 2004; Strak et al., 2012; Weichenthal et al., 2011; Kubesch et al., 2015a). Physical activity seems to have beneficial health effects, even when performed in a highly air polluted environment, but the effects are stronger in low TRAP environments (Kubesch et al., 2015a; Kubesch et al., 2015b).

Metabolomics is the systematic investigation of metabolites, with the aim of identification and quantification, within cells, biofluids, tissues or organisms. This is done on a large scale, usually at a specific point in time. It is a potentially useful approach to address the challenges associated with health effects of air pollution, in particular because air pollution is a mixture of various components, each with potentially independent effects. The molecular and biochemical pathways that link air pollution to adverse health outcomes, that are likely to vary by pollutant component, can in principle be assessed by metabolomics (Vlaanderen et al., 2017).

The aim of this work is to investigate the association between short-term exposure to traffic related air pollution in two experimental short-term studies and perturbation of metabolomic pathways, in order to shed light on the mechanisms linking TRAP exposure to adverse health outcomes. The two locations we have used represent different exposure scenarios, with Oxford Street mainly characterized by diesel vehicles, and Barcelona by normal mixed urban traffic.

## 2 Material and methods

### 2.1 Participants and samples

We conducted two experimental studies within the EXPOsOMICS consortium, the Oxford Street II study (Sinharay et al., 2017) and the Transportation, Air pollution and Physical ActivitieS (TAPAS) II study, both with a randomized crossover design. Each participant served as their own control, to exclude confounding by factors that are constant within an individual over time but vary between participants. To avoid a diurnal effect, all experiments and measurements were scheduled at the same time during the day.

#### 2.1.1 Oxford Street II (London)

Sixty participants were recruited by advertisement within the Royal Brompton hospital (cardiovascular and respiratory clinics) and local universities. Participants were divided into three groups: 1) healthy volunteers (n = 20) with a normal lung function and without a history of ischaemic heart disease (IHD); 2) patients with chronic obstructive pulmonary disease (COPD) (n = 20), without a history of IHD; and 3) patients with clinically stable IHD over the past six months (n = 20) without COPD. All current smokers or former smokers for <12 months were excluded, as well as people with high occupational levels of TRAP.

Information on age, sex, body mass index (BMI), blood pressure, distance walked, diet and medication use was collected at Royal Brompton Hospital, after which the participants were driven to the start of the experiment by electric car. For 2 h in the morning, all 60 participants walked at a steady pace along Oxford Street (a busy shopping street in London where mainly diesel-powered buses and taxis are allowed), or through the traffic-free Hyde Park. The order of the exposure location was randomized for each participant and three to eight weeks separated both sessions. The total walking distance, on predefined paths, was about six kilometers at each site and participants rested for 15 min every 30 min (Sinharay et al., 2017).

For each participant and each exposure session, three serum samples were collected – 2 h before walking, 2 h after walking and 24 h after walking – on which untargeted metabolomic analyses were performed.

Signed informed consent was provided by each participant before commencement of the experiment. The study was approved by the local Research Ethics Committee.

#### 2.1.2 TAPAS II (Barcelona)

Participants (n = 30) were healthy, non-smoking, non-medication using adults, without high occupational exposures to TRAP. In order to include a representative group of volunteers they were aged between 18 and 60 years old and balanced in terms of sex and physical activity. On each study day six participants were investigated concurrently: three performed moderate physical activity on an ergometer (intermittent 15 min cycling at 50–70% of their maximum heart rate and 15 min resting) and the other three remained at rest (seated on a chair) for 2 h. This set-up was performed in one area with low level (Barceloneta) and one with high level (Ronda Litoral) air pollution exposure. Each participant was cycling and resting in each exposure situation, ensuring the following four scenarios for each of the 30 participants: 1. Performance of physical activity in high level air pollution location; 2. Performance of physical activity in low level air pollution location; 3. Resting in high level air pollution location; 4. Resting in low level air pollution location. The order of the scenarios was randomized for each participant and a minimum of four days separated each scenario.

Information on age, sex, BMI, blood pressure, and diet was collected at ISGlobal after which the participants were driven to the start of the experiment by car. For each of the 30 participants in each of the four scenarios a serum sample was collected approximately 7 h after the exposure, resulting in 120 samples, for which untargeted metabolomic analyses were performed. Finally, measurements of noise, temperature and relative humidity were obtained in each of the four scenarios mentioned above. The Clinical Research Ethical Committee of the Parc de Salut Mar approved the study and all participants gave written informed consent prior to participation.

### 2.2 Exposure variables

#### 2.2.1 Experimental exposure

Oxford Street II – During each walking session, PM_2.5_ and PM_10_ concentrations were measured using a light scattering sensor (AM510 SidePak Personal Aerosol Monitors, TSI Ltd., MI, USA). Ultrafine particle measures were taken using a unipolar diffusion charger (Philips Aerosense NanoTracer; size range of 10–300 nm); and black carbon using an optical absorption method (microAeth Model AE51 Black Carbon aerosol monitor; AEthlabs, CA, USA; flow rate 100 ml per min). Temperature and relative humidity were electronically logged, as were noise levels (Bruel and Kjaer Type 2236 Sound level meter, Naerum, Denmark). NO_2_ concentrations were taken from a stationary monitoring site on Oxford Street repeatedly passed during walks on Oxford Street. Because no monitoring was available in Hyde Park, NO_2_ concentrations were taken from the nearest representative location sited in a school playground. More details are given in Sinharay et al. (2017).

TAPAS II – During each scenario, measurements of particulate matter (PM_10_ and PM_2.5_) and nitrogen oxides (NO_X_) were collected using a Harvard Impactor (HI) (Air Diagnostics and Engineering, USA) at a flow rate of 10 l/min and a NO_x_ analyser (2B technologies, Boulder, USA), respectively. The gravimetric analysis of air quality filter samples was conducted in a specialized laboratory according to standard operating procedures. All devices were calibrated before field work started and regular quality checks were performed throughout the sampling period according to manufacturer recommendations. In both studies, lot, laboratory, and field blanks were collected on a regular basis to validate the collected data. PM_coarse_ was calculated as PM_10_ – PM_2.5_.

### 2.3 Metabolomic analyses

The methods and procedures for sample and data processing are exactly the same as used in a companion paper on metabolomics and water contaminants (van Veldhoven et al., 2018) (see Supplementary material). In brief, untargeted metabolomics of plasma samples was performed, using a UHPLC-QTOF mass spectrometer with reversed phase column and electrospray ionization in positive polarity. This resulted in features identifiable by their mono-isotopic mass and retention time, on which the statistical analyses were performed.

### 2.4 Statistical analysis

Metabolic features that were detected in <40% of the samples were excluded and the remaining features were log transformed and imputed in case of missing data using a quantile regression approach for left-censored missing data, implemented in the imputeLCMD R package (Lazar, 2015).

Exposure concentration levels, as well as noise, temperature and relative humidity were compared at high and low exposure sites using paired *t*-tests. The Spearman correlation between the different air pollutants was calculated and visualized using heatmaps.

Mixed effect generalized least square (GLS) regression models were run to identify changes in metabolic features induced by the TRAP. Mixed effect GLS models accommodate repeated measure designs by setting the participant ID as a grouping factor and assuming an unstructured variance covariance matrix across observations per participant and time point (before, 2 h after and 24 h after the walk in the Oxford Street II study) or scenario (in the TAPAS II study). Log transformed and standardized metabolite levels (as outcome variable) were regressed against measured levels of air pollutant concentrations at both sites. Average modeled air pollution concentrations one year before the experiment were used as background or long-term exposure. Differences between measured air pollution concentrations during the experiment and the background exposure were modeled so that any significant association represented a metabolic feature whose intensity was affected by experimentally-induced change in exposure level (accounting for lagging effect). A common set of potential confounders at baseline was included in all statistical models: age, sex and BMI.

A series of sensitivity analyses were performed, assessing the robustness of our findings to outlying observations and to recent air pollution concentrations. Specifically, the metabolic features distribution was truncated at 5, 10, and 20% and the significant features were compared to the significant features resulting from the full – omics distribution analysis.

In order to assess the influence of recent air pollution concentrations on the results, modeled air pollution estimates for 24 h, 72 h and 168 h before the start of the experiments were included in the models. In addition, potential confounding by noise, temperature and relative humidity was assessed in both studies and the possibility of physical activity acting as an effect modifier (increased amount of inhaled air due to physical activity may lead to higher concentrations of inhaled TRAP) was assessed in the TAPAS II study. This was allowed by the specific study design, whereas in Oxford Street II there was not enough inter-individual variability in physical activity. Finally, stratified analyses were performed to study the effect of air pollution on the metabolome in healthy participants, compared with IHD and COPD patients (in the Oxford Street II study only).

Associations were deemed significant based on a Bonferroni corrected significance level (ensuring a family-wise error rate < 0.05), accounting for multiple testing. The overlap in the significant metabolic features was calculated and displayed in Venn diagrams and the correlation between the significant compounds was visualized using heatmaps. All analyses were performed using R version 3.1.3 (2015-03-09).

### 2.5 Annotation of metabolic features

Annotation of the discriminating features was done in four steps: 1) The LC-MS features were grouped based on retention time similarity and intensity correlation across the samples to assist in identifying ions originating from the same compound. 2) The *m*/*z* values of all the features were searched against the Human Metabolome Database (HMDB, www.hmdb.ca, as of 2nd June 2017) and Metlin (metlin.scripps.edu) using [M + H]^+^ and [M + Na]^+^ as adducts and ±8 ppm for monoisotopic mass tolerance. 3) Quality of the chromatographic peaks and spectra was inspected and the plausibility of HMDB and Metlin candidates was assessed based on retention time, isotope pattern and adduct formation. 4) Identification was confirmed by reanalysis of representative samples and pure standards when available and by comparison of the retention times and the MS/MS spectra acquired at 10V, 20V, and 40V collision energies. When standards were not available, MS/MS spectra were acquired and compared against those in mzCloud (www.mzcloud.org) or Metlin (metlin.scripps.edu). The level of identification was based on the recommendations of the Chemical Analysis Working Group of Metabolomics Standards Initiative (Sumner et al., 2007).

## 3 Results

### 3.1 Study populations and TRAP exposure

In the Oxford Street II study one participant did not complete both walks and was excluded from the analysis. An additional three participants had to be excluded because of missing values for the modeled average air pollution estimates one year before the experiment (accounting for the background levels), resulting in 56 participants with complete data.

In the TAPAS II study, two participants were excluded from the analysis due to missing metabolomic data, resulting in 28 participants with complete data. The characteristics of the study populations, stratified by group in the case of Oxford Street II, and of the two exposure sites for each study are reported in Table 1.

**Table 1:** Characteristics of study populations and exposure locations in the Oxford Street II and TAPAS II studies.

	Oxford Street II					TAPAS II
	Total (n = 56)	Healthy (n = 18)	COPD (n = 19)	IHD (n = 19)	*p*-Value⁎	Total (n = 28)
Sex – n (%)						
Male	37 (66)	9 (50)	11 (58)	17 (89)	5.791e-4	15 (54)
Female	19 (34)	9 (50)	8 (42)	2 (11)		13 (46)


	Oxford Street II					TAPAS II
	Mean ± SD	Mean ± SD	Mean ± SD	Mean ± SD	*p*-Value⁎	Mean ± SD
Age (years)	65.5 ± 6.3	64.1 ± 6.8	67.8 ± 5.4	64.4 ± 6.1	3.02e-06	40.8 ± 11.6
BMI^a^	25.0 ± 4.5	21.4 ± 3.4	25.6 ± 3.8	27.5 ± 4.0	<2e-16	24.3 ± 3.8
SBP^b^	78.7 ± 10.2	74.7 ± 8.7	89.3 ± 6.8	75.4 ± 7.9	<2e-16	–
DBP^c^	134.9 ± 19.4	136.0 ± 17.6	151.3 ± 14.7	122.4 ± 13.5	8.15e-16	–


Exposure sites	Hyde Park		Oxford Street		*p*-Value⁎⁎	Barceloneta		Ronda		*p*-Value⁎⁎
	Mean ± SD	Range	Mean ± SD	Range		Mean ± SD	Range	Mean ± SD	Range	
Noise	73.3 ± 3.6	61.4–78.0	76.2 ± 1.9	71.4–81.2	1.434e-15	60.6 ± 1.3	59.2–63.1	78.2 ± 0.9	77.1–80.5	<2.2e-16
Temperature (°C)	17.2 ± 6.7	2.6–27.3	20.1 ± 7.7	2.3–31.4	0.00042	11.9 ± 2.4	9.4–16.7	11.3 ± 3.0	7.6–18.0	0.1906
Humidity	53.5 ± 13.0	27.9–87.5	48.4 ± 17.2	9.4–81.0	0.0024	56.5 ± 13.8	27.4–80.3	55.7 ± 10.3	42.6–73.5	0.7039

⁎ *p*-Values are for comparisons according to the group (healthy, COPD, IHD).

⁎⁎ *p*-Values are for the comparison according to location (Hyde Park vs Oxford Street and Barceloneta vs Ronda).

a Body Mass Index is calculated as the weight in kilograms divided by the square of the height in meters.

b Systolic blood pressure.

c Diastolic blood pressure.

**Fig. 1:**
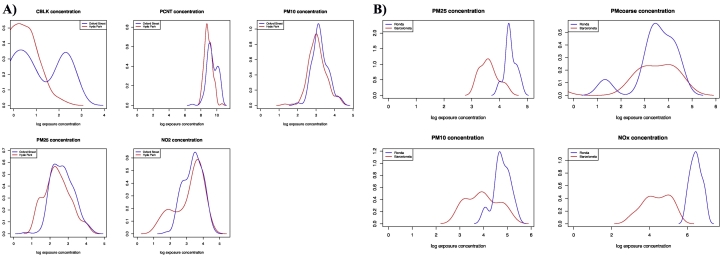
Air pollution concentrations at both locations in the Oxford Street II cohort (A) and TAPAS II cohort (B).

The exposure concentrations at the two locations during the experiment are reported in Table 2 and Fig. 1.

**Table 2:** Description of exposures at both locations, in the Oxford Street II and TAPAS II studies.

	Oxford Street II						
	Hyde Park			Oxford Street			*p*-Value⁎
	N	Mean ± sd	Range	n	Mean ± sd	Range	
Exposure^a^ (unit)							
CBLK (μg/m^3^)	47	1.7 ± 1.2	0.3–5.2	49	10.9 ± 3.1	6.7–17.8	<2.2e-16
NO_2_ (μg/m^3^)	41	10.3 ± 7.7	3.0–40.5	38	17.3 ± 7.6	6.1–46.2	1.3e-06
PM_2.5_ (μg/m^3^)	50	11.2 ± 13.0	3.4–60.8	49	20.5 ± 12.9	6.4–75.5	0.0005
PM_10_ (μg/m^3^)	50	23.0 ± 16.4	3.7–80.2	49	32.4 ± 14.7	14.1–84.4	0.001
UFP (particles/cm^3^)	50	6738 ± 3268.8	2846–15,690	48	24,840 ± 8087.6	7197–38,640	<2.2e-16


	TAPAS II						
	Barceloneta			Ronda			*p*-Value⁎
	N	Mean ± sd	Range	n	Mean ± sd	Range	
PM_2.5_ (μg/m^3^)	28	39.2 ± 13.7	24.9–68.8	29	82.5 ± 17.1	54.7–116.3	1.025e-11
PM_10_ (μg/m^3^)	28	64.1 ± 41.9	22.4–144.8	29	124.6 ± 40.5	58.4–204.0	9.117e-06
PM_coarse_ (μg/m^3^)	28	25.6 ± 28.5	0.0–75.9	29	42.1 ± 25.0	3.8–87.7	0.04916
NO_x_ (ppb)	28	99.3 ± 61.9	22.9–212.8	29	660.8 ± 119.7	389.4–1063.0	2.083e-13

a CBLK = black carbon, NO_2_ = nitrogen dioxide, PM_2.5_ = particulate matter of 2.5 μm or less in diameter, PM_10_ = particulate matter of 10 μm or less in diameter, UFP = ultra-fine particles, PM_coarse_ = PM_2.5_ - PM_10_, NO_x_ = nitrogen oxides.

⁎ *p*-Values are for pair-wise comparisons according to the location (Hyde Park vs Oxford Street and Barceloneta vs Ronda).

Although the tail ends of the exposure distributions overlap, overall exposures are significantly higher in Oxford Street compared with Hyde Park (especially for CBLK and UFP) and in Ronda compared with Barceloneta (especially for PM_2.5_, PM_10_ and NO_x_). There were considerably higher air pollution levels in the TAPAS II study compared with the Oxford Street II study. Correlations were observed between most of the air pollution exposures in both the Oxford Street II study (Supplementary Fig. S1) and in the TAPAS II study (Supplementary Fig. S2), especially between PM_2.5_ and PM_10_.

### 3.2 Metabolic profiles

After imputation, a total of 5794 and 7810 metabolic features were included in the analyses of the Oxford Street II study and TAPAS II study, respectively. Truncation of the – omics distribution showed that our results are robust (see Supplementary Table S3). As can be seen in the Supplementary material, associations were not confounded by other exposures such as noise, temperature, humidity (see S4 and Supplementary Table S4), past exposures (24 h, 72 h, 168 h before the experiment) (see S3 and Supplementary Fig. S3) or disease status (in Oxford Street II) (see S5 and Supplementary Table S6), and there was no indication of physical activity acting as an effect modifier or confounder in either study (see S4 and Supplementary Table S5).

#### 3.2.1 Oxford Street II

Our GLS model, adjusted for age, sex, BMI, group (healthy, IHD, COPD) and caffeine intake (yes/no), identified one, two, one and 27 features that were associated with concentrations of PM_2.5,_ PM_10_, CBLK and NO_2,_ respectively. All these significant features can be found in Supplementary Table S1.Out of the 27 features associated with NO_2_, two were also associated with COPD.

**Fig. 2:**
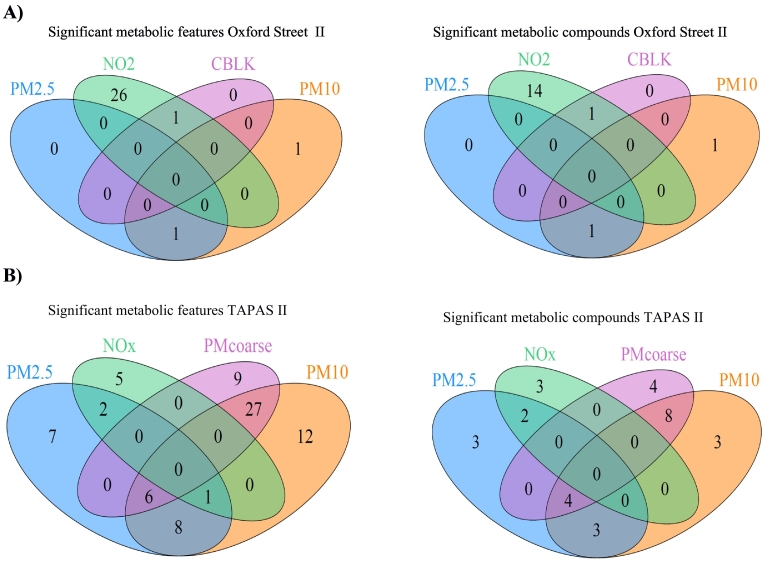
Venn diagram of overlapping significant metabolomic features and overlapping significant metabolic compounds for each pollutant for the Oxford Street II study (A) and TAPAS II study (B).

**Fig. 3:**
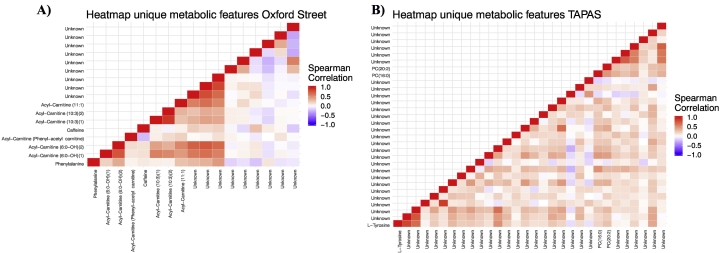
Heatmap of Spearman correlations between the unique metabolic compounds associated with single pollutant concentrations in the Oxford Street II (A, 17 compounds) and TAPAS II (B, 30 compounds) study.

The Venn-diagram in Fig. 2A shows there is one feature associated with both NO_2_ and CBLK concentrations while another feature is associated with both PM_2.5_ and PM_10_ concentrations. All other features are uniquely associated with only one exposure, based on Bonferroni statistical significance. The second Venn-diagram displays the overlap between the significantly associated compounds. The metabolomic compounds for which higher reciprocal correlations are observed were all associated with NO_2_ (see Fig. 3A). The results of the stratified analysis by health status (healthy, COPD, IHD) can be found in the Supplementary materials (S5 and Supplementary Table S4).

#### 3.2.2 TAPAS II

The GLS model for the TAPAS II study was adjusted for age, sex, BMI and physical activity and identified 24, 54, 42 and eight features that were associated with concentrations of PM_2.5_, PM_10_, PM_coarse_ and NO_X_ respectively (see Supplementary Table S2). Out of all metabolic features associated with PM_2.5_, PM_10_ and NO_X_, none were associated with physical activity.

The Venn-diagram in Fig. 2B shows there is some overlap in the significant metabolic features associated with the PMs (PM_2.5_, PM_10_, PM_coarse_). However, only three features are associated with both NO_X_ and PM_2.5_, one with both NOx and PM_10_ and six features with PM_coarse_, PM_2.5_ and PM_10_ concentrations, while all other features are uniquely associated with the various pollutants. The significantly associated compounds are displayed in the second Venn-diagram of Fig. 2B. The heatmap of the correlation between the significantly associated metabolic compounds (Fig. 3B) again shows little correlation, similar to what was observed in the Oxford Street II study.

### 3.3 Annotation of metabolic profiles

Some of the 29 and 77 features that were found to be associated with TRAP in both studies belong to the same compound. After grouping these features based on identical retention time and intensity correlations, the numbers of features are reduced to 17 and 30 compounds for the Oxford Street II and TAPAS II study, respectively.

#### 3.3.1 Oxford Street II

Annotation was carried out for the 17 compounds associated with at least one air pollutant. Two compounds were identified as phenylalanine and caffeine (see Table 3). Six of the compounds were annotated as acyl-carnitines with different carbon chain lengths and insaturations. The remaining compounds were classified as “unknown” either because there was no database match or there was not enough evidence to support a match from HMDB (usually when the signal was too weak to be fragmented or when the signal did not give a specific enough fragmentation spectrum).

**Table 3:** Compounds (N = 17) associated with at least one traffic related air pollutant in the Oxford Street II study (sorted on mass to charge ratio).

Identity	*m*/*z* (Da)	Retention time (min)	*m*/*z* difference (ppm)	Identification level^a^	Direction	Associated pollutant
Phenylalanine	166.0868	2.04	−2.9	1	Down	NO_2_
Caffeine	195.0877	3.19	1.1	1	Up	NO_2_
Acyl-carnitine (6:0-OH) (1)	276.1805	2.22	0.1	3	Down	NO_2_
Acyl-carnitine (6:0-OH) (2)	276.1805	2.4	0.4	3	Down	NO_2_
Unknown	276.1993	5.42		4	Down	NO_2_
Acyl-carnitine (phenyl-acetyl carnitine)	280.1543	2.76	1.2	3	Down	NO_2_
Unknown	290.1504	7.26		4	Down	NO_2_
Unknown	302.215	5.66		4	Down	NO_2_
Unknown	309.1515	7.19		4	Down	CBLK
					Up	NO_2_
Acyl-carnitine (10:3) (1)	310.2013	4.17	1.7	3	Down	NO_2_
Acyl-carnitine (10:2) (2)	310.2013	4.35	1.4	3	Down	NO_2_
Acyl-carnitine (11:1)	328.2482	5.12	0.7	3	Down	NO_2_
Unknown	330.2465	5.94		4	Down	NO_2_
Unknown	407.2763	7.18		4	Down	PM_2.5_
					Up	PM_10_
Unknown	443.4101	8.54		4	Up	NO_2_
Unknown	461.2413	7.45		4	Down	PM_10_
					Down	PM_2.5_
Unknown	624.6289	7.09		4	Up	NO_2_

Level 1 (identity confirmed): retention time and MS/MS matched with an authentic chemical standard; Level 3 (putatively characterized compound classes): no standard available or analyzed but mass within 5 ppm mass error and MS/MS spectra matches with those in a database.

a Identification level as defined by Sumner et al. (2007).

#### 3.3.2 TAPAS II

Annotation was carried out for the 30 compounds associated with a least one air pollutant (Table 4). Among these, l-Tyrosine and two PCs were identified as associated with at least one particulate matter (PM) exposure variable. Polyethylene Glycols (PEGs) were excluded from the results, as they are most likely due to pre-analytical and analytical factors. The remaining compounds were classified as unknown.

**Table 4:** Compounds (N = 30) associated with at least one traffic related air pollutant in the TAPAS II study (sorted on mass to charge ratio).

Identity	*m*/*z* (Da)	Retention time (min)	*m*/*z* difference (ppm)	Identification level^a^	Direction	Associated pollutant
Unknown	175.1114	6.17		4	Up	PM_2.5_, PM_10_
l-Tyrosine	182.0812	1.34	0.1	1	Up	PM_coarse_, PM_10_
Unknown	229.1171	2.48		4	Up	PM_2.5_, PM_10_
Unknown	233.1158	5.07		4	Up	NO_x_
Unknown	239.162	5.72		4	Up	PM_2.5_, NO_x_
Unknown	263.1392	3.13		4	Up	PM_coarse_, PM_2.5_, PM_10_
Unknown	267.1329	1.87		4	Up	PM_coarse_
Unknown	271.0866	5.72		4	Up	NO_x_
Unknown	283.2419	6.8		4	Up	PM_coarse_
Unknown	302.1961	2.13		4	Up	PM_coarse_
Unknown	302.1963	1.86		4	Up	PM_coarse_, PM_10_
Unknown	309.2281	4.98		4	Up	PM_10_
Unknown	319.224	6.67		4	Up	PM_2.5_, NO_x_
Unknown	346.1044	1.36		4	Up	PM_10_
Unknown	351.1547	4.65		4	Up	PM_2.5_, PM_10_
Unknown	362.0995	1.35		4	Up	PM_coarse_, PM_10_
Unknown	367.1935	4.81		4	Up	PM_2.5_
Unknown	367.3287	7.7		4	Up	PM_coarse_, PM_10_
Unknown	387.1958	6.17		4	Up	PM_coarse_, PM_10_
Unknown	397.1992	6.72		4	Up	PM_coarse_
Unknown	421.251	6.79		4	Up	PM_coarse_, PM_2.5_, PM_10_
Unknown	453.2453	2.69		4	Up	PM_coarse_, PM_10_
Unknown	471.7381	3.8		4	Up	NO_x_
PC (16:0)	502.3284	7.14	−3.1	3	Up	PM_2.5_, PM_10_
PC (20:2)	570.3553	7.16	0.2	3	Up	PM_2.5_
Unknown	575.4643	7.69		4	Up	PM_10_
Unknown	581.4497	8.13		4	Up	PM_coarse_, PM_10_
Unknown	612.5152	7.54		4	Up	PM_coarse_, PM_2.5_, PM_10_
Unknown	617.4728	7.5		4	Up	PM_coarse_, PM_2.5_, PM_10_
Unknown	784.527	7.79		4	Up	PM_coarse_, PM_10_

Level 1 (identity confirmed): retention time and MS/MS matched with an authentic chemical standard; Level 3 (putatively characterized compound classes): no standard available or analyzed but mass within 5 ppm mass error and MS/MS spectra matches with those in a database.

a Identification level as defined by Sumner et al. (2007).

The MS/MS spectra of the identified compounds in both studies can be found in the Supplementary materials (S6).

## 4 Discussion

This study, together with (Vlaanderen et al., 2017; Ladva et al., 2017), represents one of the first agnostic projects investigating the effect of short-term exposure to traffic-related air pollutants (TRAP) on the metabolome, utilizing two experimental studies with a crossover design, alternating higher and lower TRAP. The two studies represent two different exposure scenarios, mainly diesel vehicles in London and mixed urban traffic in Barcelona. We identified various statistically significant associations between air pollution concentrations and levels of metabolic features in blood. More associations were found in the TAPAS II study compared with the Oxford Street II study, possibly due to the higher exposure levels and greater variability between sites in Barcelona. Remarkably, in both studies little overlap was observed between the metabolite levels associated with different air pollutants, although some overlap was observed in the features associated with the different PMs, suggesting unique effects of the various pollutants in the air pollution mixture. This observation needs replication in other settings but may lead to the identification of pollutant-specific metabolic pathways in the TRAP mixture. Associations were not confounded by other exposures such as noise, temperature, humidity, past exposures, disease status (in Oxford Street II) or physical activity.

Observing the annotated metabolic compounds associated with TRAP concentrations we aimed to identify perturbed metabolic pathways. Results showed levels of various acyl-carnitines (Table 3) to be affected by air pollution concentrations in Oxford Street II, mostly in association with NO_2_. We observed a positive dose-response relationship between levels of three acyl-carnitines with levels of air pollution, while three other acyl-carnitines decreased with higher levels of air pollution. Notably, out of the 15 compounds associated with NO_2_, two were also associated with COPD.

Acyl-carnitines are metabolites that are involved in fatty acid oxidation and are known to play a role in energy metabolism in cardiac tissue (Makrecka et al., 2014). Acyl-carnitines and LysoPCs were also found associated with NO_2_ in two previous Exposomics studies nested in the SAPALDIA and EPIC cohorts (Jeong et al., 2018). In addition, several studies have shown altered levels of acyl-carnitines in relation to inspiratory muscle weakness (Kilicli et al., 2010), emphysema (Conlon et al., 2016) and COPD (Suleman et al., 2013). However, acyl-carnitines did not emerge as significant in TAPAS II, and in general there was no overlap between the two studies for the annotated features. Perturbation in phenylalanine pathways have been previously associated with urban traffic exposures in (Pradhan et al., 2016).

In conclusion, we used two experimental crossover studies corresponding to two different exposure scenarios (diesel vehicles in London and mixed urban traffic in Barcelona). The main result was the association of different metabolomic features with different pollutants, with most associations identified for NO_2_ in Oxford Street II and particulate matter in TAPAS II.

## Declaration of competing financial interests

The authors declare they have no actual or potential competing financial interests.

## Appendix A Supplementary data

Supplementary material Image 1
