# Supplementary material for: Impact of short-term traffic-related air pollution on the metabolome – Results from two metabolome-wide experimental studies
Source: Environ Int. 2019 Feb;123:124–31. doi: 10.1016/j.envint.2018.11.034 (PMC6329888; doi:10.1016/j.envint.2018.11.034)
Supplement: Supplementary file 1 — Supplementary material [file mmc1.docx]

**Supplementary Material**

*S1. Sample processing*

Samples were prepared by mixing 20 µL of plasma with 150 µL of acetonitrile and vacuum filtered into polypropylene well plates that were sealed until analysis (Captiva ND 0.2 μm filter and collection plates, Agilent Technologies, Santa Clara, CA; EPS well plate seals, BioChromato, San Diego, CA). After randomization into the batch, samples were analyzed as a single uninterrupted batch with liquid chromatography–mass spectrometry system consisting of a 1290 Binary LC, a Jet Stream electrospray ionization (ESI) source, and a 6550 QTOF mass spectrometer (Agilent Technologies, Santa Clara, USA). Autosampler tray was kept refrigerated at 5 °C and 1 µL (Oxford Street II) and 3 µL (TAPAS II) of the sample solution was injected on an ACQUITY UPLC HSS T3 column (2.1 × 100mm, 1.8 µm; Waters). Column temperature was 45 °C and mobile phase flow rate 0.4 ml/min, consisting of ultrapure water and LC-MS grade methanol, both containing 0.1% (v/v) of formic acid. The gradient profile was as follows: 0–6 min: 5% → 100% methanol, 6–10.5 min: 100% methanol, 10.5–13.5 min: 5% methanol, with the first minute diverted to waste. The mass spectrometer was operated in positive polarity using following conditions: drying gas (nitrogen) temperature 175 °C and flow 12 L/min, sheath gas temperature 350 °C and flow 12 L/min, nebulizer pressure 45 psi, capillary voltage 3500 V, nozzle voltage 300 V, and fragmentor voltage 175 V. Data was acquired using extended dynamic range mode across a mass range of 50–1200, with an acquisition rate of 1.67 Hz. Continuous mass axis calibration was performed with two reference ions (*m/z* 121.050873 and *m/z* 922.009798). The analytical run was initiated with priming injections of a pooled quality control (QC) sample to achieve stable instrument response, followed by study samples that were intervened after every 10 injections with the same pooled QC sample and a solvent blank to monitor instrument performance.

*S2. Data processing*

Pre-processing of the acquired data was performed using Qualitative Analysis B.06.00 SP1, DA Reprocessor, and Mass Profiler Professional 12.1 software (Agilent Technologies, Santa Clara, USA). The initial processing was performed using find by molecular feature (MFE) algorithm. Threshold values for mass and chromatographic peak heights were 500 and 5000 counts, respectively, with a compound quality score threshold at 70. Only singly charged ions were included. The resulting features were combined into a single list using 0.1 min retention time window for alignment, and those existing in at least 2% of all the samples were used as targets for a recursive feature extraction of the raw data using a find by formula (FBF) algorithm. For the recursive process, match tolerances were ±15 ppm and ±0.15 min for the mass and retention time for Oxford Street II and and ±20 ppm and ±0.20 min for TAPAS II. Ion species were limited to [M+H]^+^ , [M+Na]^+^, [M-H_2_O+H]^+^ and [M]^+^, without filtering by peak height or quality score.

*S3. Sensitivity analysis*

A series of sensitivity analyses were performed, assessing the robustness of our findings to outlying observations and to recent air pollution concentrations. Specifically, the metabolic features distribution was truncated at 5, 10, and 20% and the significant features were compared to the significant features from the full –omics distribution. Supplementary Table S3 shows a similar number of significant associations for each air pollutant, which indicates that these findings are robust and unlikely due to outlying observations.

In order to assess the influence of recent air pollution concentrations on the results, average air pollution estimates at 24h, 72h and 168h before the start of the experiments were included in the models. These modelled estimates were obtained using London Air Quality Network monitoring sites in the Oxford Street II study. Assessment was based on the participant’s home or work address and was calculated by multiplying the annual exposure estimate for their address in the year 2013 by a temporal factor derived from the ratio of measured concentration for that period divided by the annual mean measured concentration (Beevers, 2012, Atmost Environ). In the TAPAS II study assessment was also based on the participant’s home address and used time-adjusted Land Use Regression (LUR) models, estimating mean exposure concentrations for the year 2009 (Beelen, 2013, Atmospheric Environment).

The number of associations for the various models and the overlap in associated features is displayed in the Venn diagram in Figure S3A for Oxford Street II and S3B for TAPAS II.

For Oxford Street II it shows that the number of associations found for the various models including recent air pollution concentrations (35, 32, 34 significant features for the model including 24h, 72h and 168h respectively) is very similar to the 29 features identified in the model without recent air pollution exposure. In addition, the overlap in features is large which means that recent air pollution concentrations do not seem to affect the results found to be associated with the experiment. In the TAPAS II study however, when including 24h, 72h and 168h exposure measurements, there were some convergence problems for the PMcoarse exposure due to a large number of missing values (36% for PMcoarse at 24h, 45% for PMcoarse at 72h and 27% for PMcoarse at 168h). Due to too many missing values per scenario, this inflated the number of significant features to 988, 1,012 and 1,015 for 24h, 72h and 168h respectively. Therefore, these results for the TAPAS II study cannot be interpreted.

*S4. Confounding and interaction*

Adjustment for the potential confounding factors noise, temperature and humidity inflated the number of associations found in both the Oxford Street II and the TAPAS II study. In the Oxford Street II study, a clear association was seen between these variables and exposure. However, very few metabolic features were significantly associated with these variables (Supplementary Table S4) and the ones that were associated did not overlap with the significant features identified in the final model. In the TAPAS II study, noise was significantly associated with the exposure site, while temperature and relative humidity were not, therefore the latter two were not included as potential confounders. Nine features were significantly associated with noise in the model for PM_2.5_, three in the model for PM_coarse_ and 14 in the model for NO_x_. However, none of these hits overlapped with the significant features identified in the final model.

The possibility of physical activity acting as an effect modifier (increased amount of inhaled air due to physical activity may lead to higher concentrations of inhaled TRAP) was assessed in the TAPAS II study. The interaction term between physical activity and exposure concentrations was not significant for any of the air pollutants. The number of metabolomic hits significantly associated with exposure in the model including the interaction term was lower than in the model without the interaction term (see Supplementary Table S5). With one exception, all features found to be associated with exposure concentrations in the model including the interaction term were also found in the model without the interaction term.

*S5. Health status*

Stratified analysis by disease status, in the Oxford Street II study only, showed a difference in the number of significant signals in healthy individuals vs individuals with COPD and IHD (Supplementary Table S6). In the IHD group, associations with PM_10_ and PM_2.5_ were detected, but not with other pollutants due to convergence issues. COPD patients showed fewer significant associations with PM_10_, PM_2.5_ and NO_2_ and more with CBLK with respect to healthy individuals, while IHD patients showed more associations when compared to the other groups. When comparing the healthy individuals and the patients with either COPD or IHD, fewer associations were detected in all exposures but CBLK, where the numbers of significant association were very close in the two groups.

**SUPPLEMENTARY FIGURES**

**Supplementary Figure S1.** Heatmap of correlation between air pollution concentrations at Hyde Park (S1-A) and at Oxford Street (S1-B)

**S1-A**


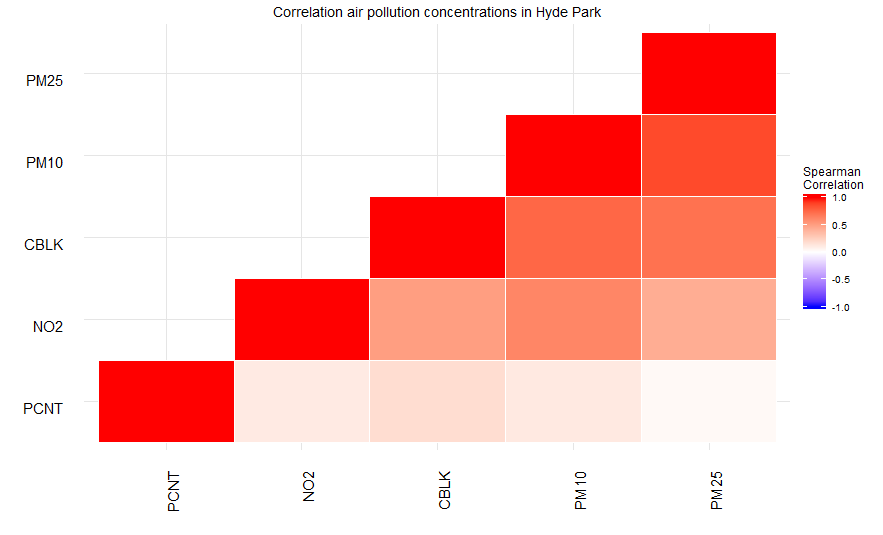


**S1-B**


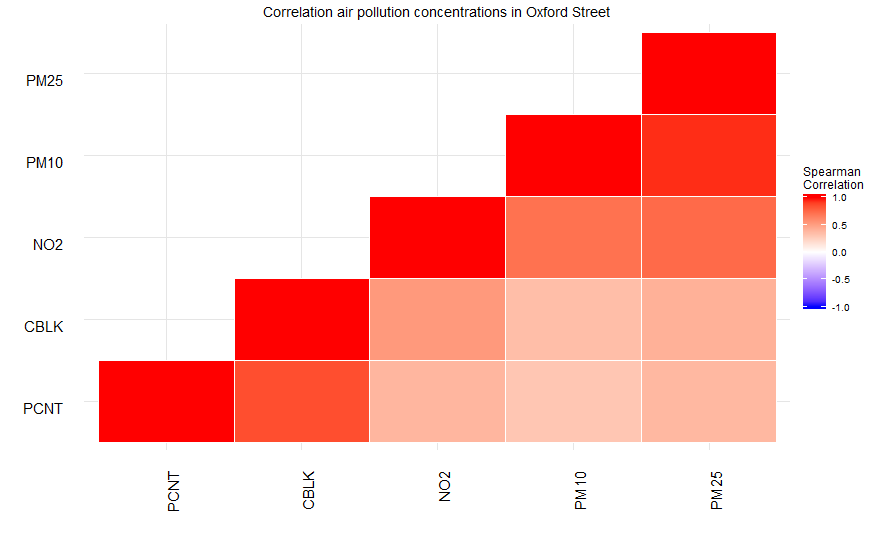


**Supplementary Figure S2.** Heatmap of correlation between air pollution concentrations at Barceloneta (S2-A) and at Ronda (S2-B)

**S2-A**


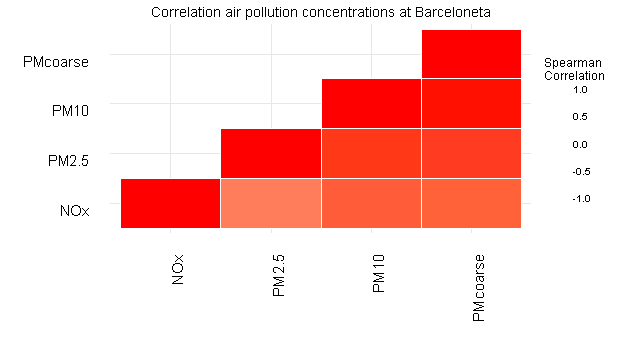


**S2-B**


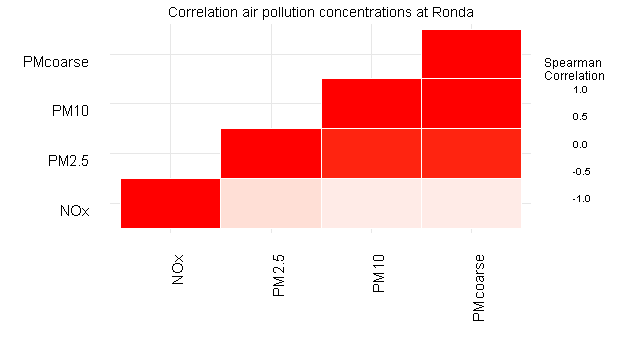


Supplementary Figure S3. Venn diagram of overlapping metabolic features for the final model (excluding recent exposure concentrations) and the models including recent exposure concentrations for the Oxford Street II study (A) and TAPAS II study (B). Recent exposures included concentrations measured 24h, 72h and 168h prior to the experiment.

**A.**


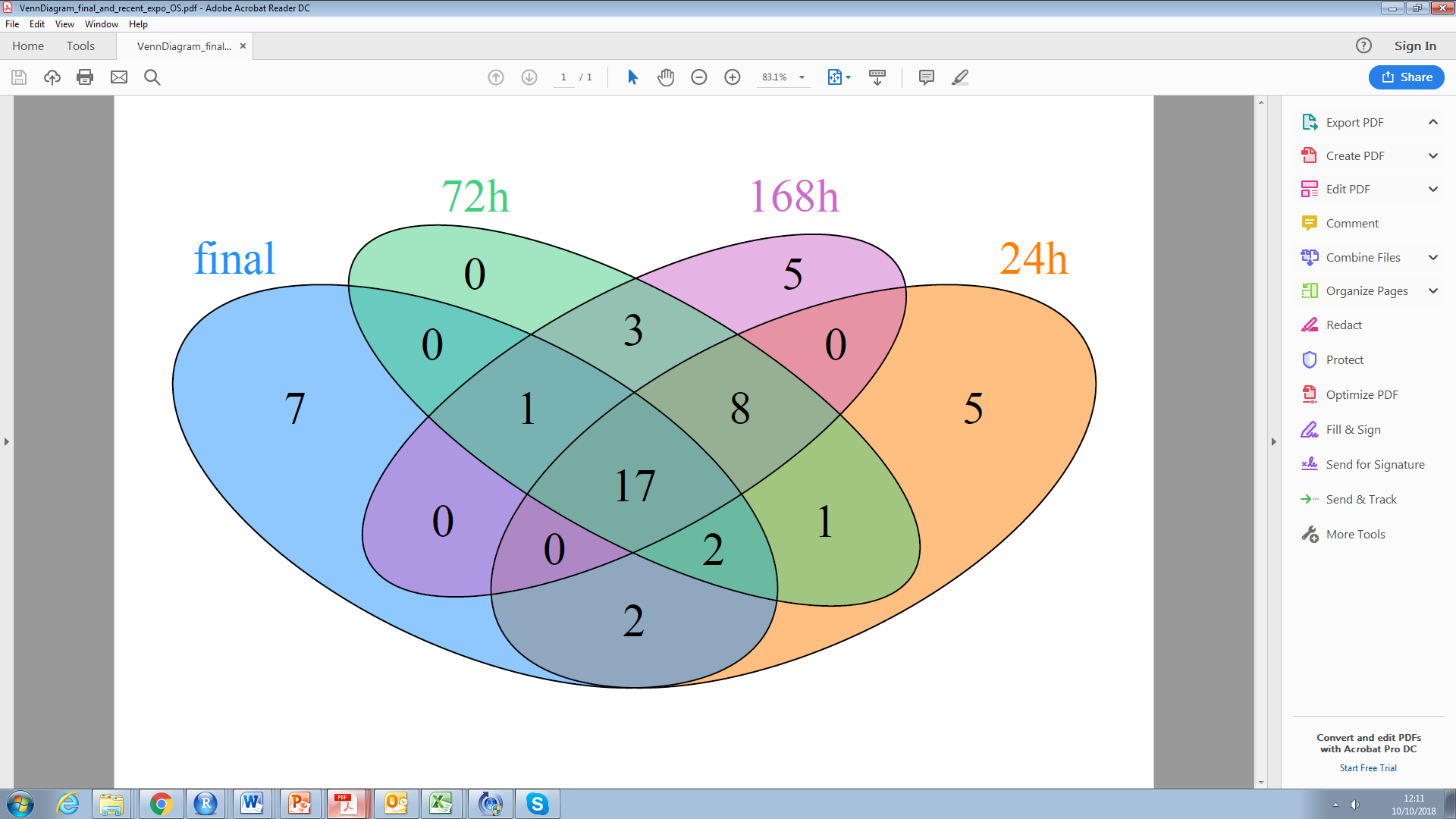


**B.**


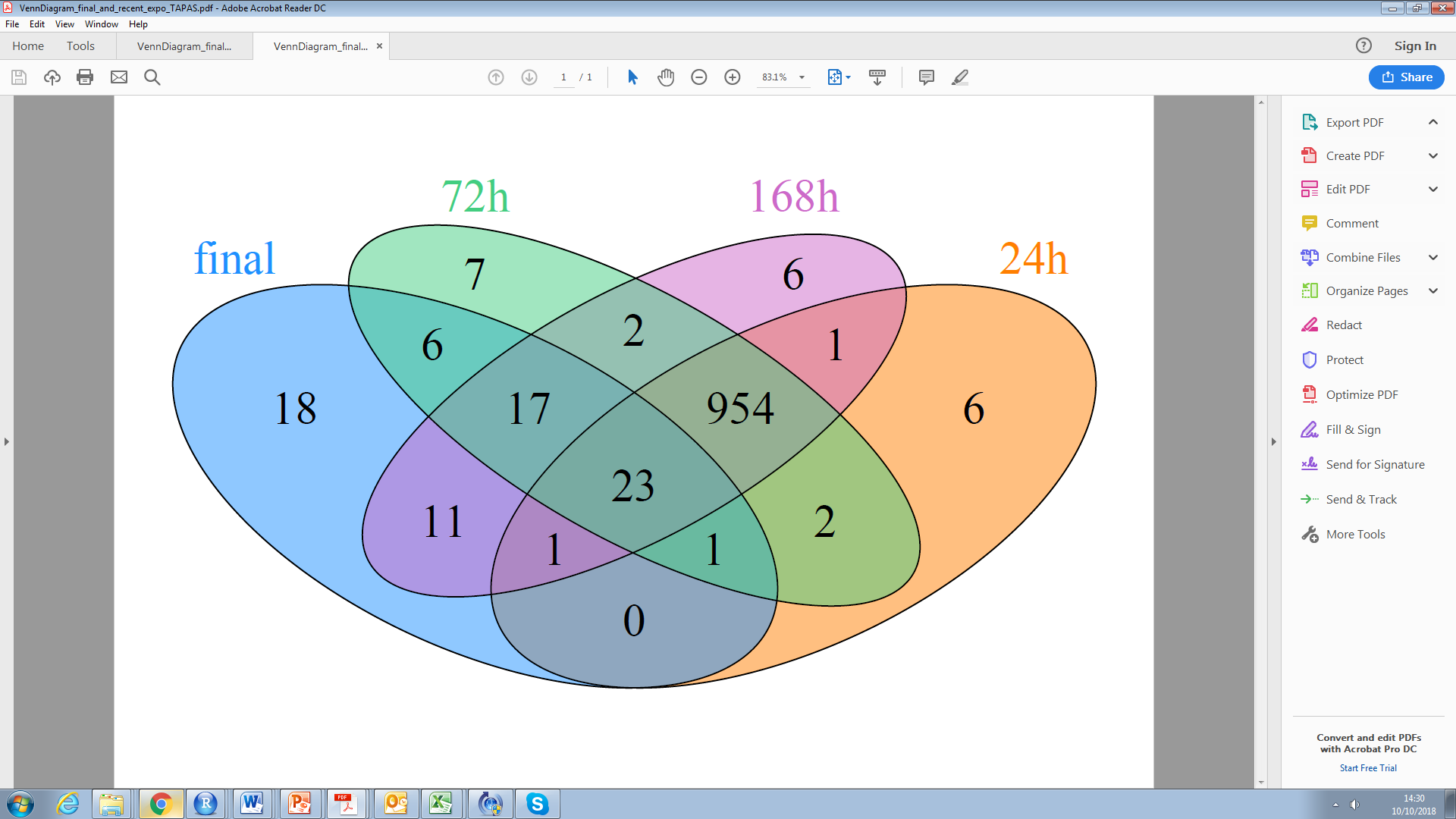


**SUPPLEMENTARY TABLES**

**Supplementary Table S1.** Total number of mass spectrometry metabolomic features (mono-isotopic mass @ retention time) significantly associated with each air pollutant after Bonferroni correction for multiple testing, in the Oxford Street II study

| **PM_2.5_** | **PM_10_** | **CBLK** | **NO_2_** |
| --- | --- | --- | --- |
| 460.234@7.450546 | 406.269@7.1838274 | 308.1442@7.197069 | 102.0467@2.046496 |
|  | 460.234@7.450546 |  | 102.0469@2.045386 |
|  |  |  | 119.0738@2.0438619 |
|  |  |  | 134.0727@2.0469444 |
|  |  |  | 165.0795@2.0396664 |
|  |  |  | 165.0795@2.0465238 |
|  |  |  | 166.0825@2.0461037 |
|  |  |  | 194.0802@3.189687 |
|  |  |  | 216.0619@3.190204 |
|  |  |  | 275.1731@2.4011953 |
|  |  |  | 275.1732@2.2214637 |
|  |  |  | 275.192@5.422026 |
|  |  |  | 279.1467@2.756788 |
|  |  |  | 289.1431@7.261201 |
|  |  |  | 296.0202@2.0461934 |
|  |  |  | 301.2068@5.671208 |
|  |  |  | 301.2077@5.6644535 |
|  |  |  | 308.1442@7.197069 |
|  |  |  | 309.1935@4.170545 |
|  |  |  | 309.1936@4.3509645 |
|  |  |  | 325.0236@3.1906562 |
|  |  |  | 327.2407@5.1196 |
|  |  |  | 329.2392@5.940575 |
|  |  |  | 386.0776@2.0465972 |
|  |  |  | 442.4028@8.540412 |
|  |  |  | 623.6216@7.09019 |
|  |  |  | 633.2764@7.2653646 |

**Supplementary Table S2.** Total number of mass spectrometry metabolomic features (mono-isotopic mass @ retention time) significantly associated with each air pollutant after Bonferroni correction for multiple testing, in the TAPAS II study

| **PM_2.5_** | **PM_10_** | **PM_coarse_** | **NO_x_** |
| --- | --- | --- | --- |
| 130.9667@1.1605076 | 130.9667@1.1605076 | 135.0684@1.3379543 | 194.1152@2.3479009 |
| 174.1041@6.169871 | 135.0684@1.3379543 | 164.047@1.3391057 | 232.1085@5.0674195 |
| 181.183@3.6024098 | 164.047@1.3391057 | 181.0739@1.3382499 | 238.1547@5.7245855 |
| 194.115@1.8453895 | 174.1041@6.169871 | 228.1098@2.4842267 | 270.0793@5.724411 |
| 194.1152@2.3479009 | 181.0739@1.3382499 | 262.1319@3.1287959 | 318.2167@6.6679406 |
| 216.0975@2.3479764 | 181.183@3.6024098 | 266.1256@1.8709315 | 470.7308@3.8021672 |
| 217.9878@2.7767053 | 194.115@1.8453895 | 282.2346@6.803709 | 614.2425@5.105483 |
| 219.9885@2.7766294 | 194.1152@2.3479009 | 301.1888@2.1274965 | 630.2673@4.325337 |
| 228.1098@2.4842267 | 216.0975@2.3479764 | 301.189@1.862923 |  |
| 238.1547@5.7245855 | 219.9885@2.7766294 | 361.0922@1.349559 |  |
| 239.0003@4.2785835 | 228.1098@2.4842267 | 362.3022@7.0567937 |  |
| 262.1319@3.1287959 | 238.1427@2.4161224 | 366.3214@7.70186 |  |
| 318.2167@6.6679406 | 262.1319@3.1287959 | 370.2189@3.2990222 |  |
| 329.2195@4.199553 | 301.189@1.862923 | 379.3294@7.056878 |  |
| 342.1286@3.9527452 | 308.2208@4.9836965 | 386.1885@6.1657004 |  |
| 350.1474@4.654804 | 326.1945@3.0691373 | 396.1919@6.723462 |  |
| 366.1862@4.812915 | 334.1285@3.6529694 | 400.2619@6.782114 |  |
| 420.2437@6.7902265 | 345.0971@1.357729 | 406.3288@7.0582557 |  |
| 452.7405@2.6863506 | 350.1474@4.654804 | 407.3604@7.32363 |  |
| 501.3211@7.1377435 | 361.0922@1.349559 | 420.2437@6.7902265 |  |
| 569.348@7.1576624 | 362.3022@7.0567937 | 423.3555@7.057786 |  |
| 611.5079@7.540714 | 366.3214@7.70186 | 423.3558@7.0576344 |  |
| 611.5106@7.5401154 | 370.2189@3.2990222 | 428.311@7.0583997 |  |
| 616.4664@7.509964 | 370.2191@3.4275684 | 450.3549@7.0580688 |  |
|  | 379.3294@7.056878 | 451.3867@7.320985 |  |
|  | 386.1885@6.1657004 | 451.3869@7.320985 |  |
|  | 400.2619@6.782114 | 467.3813@7.0580125 |  |
|  | 406.3288@7.0582557 | 467.3818@7.058089 |  |
|  | 420.2437@6.7902265 | 478.3852@7.3173275 |  |
|  | 423.3555@7.057786 | 494.3805@7.057227 |  |
|  | 423.3558@7.0576344 | 511.4081@7.0573316 |  |
|  | 428.311@7.0583997 | 511.4082@7.0573316 |  |
|  | 451.3867@7.320985 | 516.3491@6.7559314 |  |
|  | 451.3869@7.320985 | 538.4069@7.055746 |  |
|  | 467.3813@7.0580125 | 539.4392@7.312478:1 |  |
|  | 467.3818@7.058089 | 539.4392@7.312478:2 |  |
|  | 468.2779@7.755583 | 580.4424@8.133824 |  |
|  | 494.3805@7.057227 | 583.4653@7.307085 |  |
|  | 501.3211@7.1377435 | 611.5079@7.540714 |  |
|  | 511.4081@7.0573316 | 611.5106@7.5401154 |  |
|  | 511.4082@7.0573316 | 616.4664@7.509964 |  |
|  | 522.4115@7.3119335 | 783.5197@7.790163 |  |
|  | 539.4392@7.312478:1 |  |  |
|  | 539.4392@7.312478:2 |  |  |
|  | 574.457@7.6932445 |  |  |
|  | 580.4424@8.133824 |  |  |
|  | 583.4653@7.307085 |  |  |
|  | 611.5079@7.540714 |  |  |
|  | 611.5106@7.5401154 |  |  |
|  | 616.4655@7.5031905 |  |  |
|  | 616.4664@7.509964 |  |  |
|  | 636.2602@6.8680887 |  |  |
|  | 674.5254@7.481764 |  |  |
|  | 783.5197@7.790163 |  |  |

Supplementary Table S3. Truncation of the -omics distribution - number of mass spectrometry metabolomic features significantly associated with each air pollutant after Bonferroni correction for multiple testing, in the Oxford Street II and TAPAS II studies.

|  | **Number of associations^a^** | | | | | | | | |
| --- | --- | --- | --- | --- | --- | --- | --- | --- | --- |
|  | **OXFORD STREET II** | | | | | **TAPAS II** | | | |
| **Omics distribution** | **CBLK** | **NO_2_** | **PM_10_** | **PM_2.5_** | **UFP** | **PM_2.5_** | **PM_10_** | **PM_coarse_** | **NO_x_** |
| >5% and <95% | 1 | 15 | 2 | 1 | 0 | 13 | 19 | 16 | 5 |
| >10% and <90% | 1 | 18 | 0 | 1 | 0 | 24 | 33 | 26 | 12 |
| >20% and <80% | 1 | 15 | 2 | 2 | 0 | 15 | 24 | 24 | 9 |

^a^ Statistical models are adjusted for age, sex, BMI with the addition of group (healthy, COPD, IHD) in the Oxford Street II study and physical activity (yes/no) in the TAPAS II study

**Supplementary Table S4.** Number of mass spectrometry features significantly associated with each air pollutant in the final model and the number of mass spectrometry features significantly associated with noise, temperature and humidity in the additionally adjusted models, after Bonferroni correction for multiple testing, in the Oxford Street II study.

|  | **Final model** | **Noise** | **Temperature** | **Humidity** |
| --- | --- | --- | --- | --- |
| **CBLK** | 2 | 7 | 244 | 54 |
| **NO**_2_ | 36 | 3 | 21 | 4 |
| **PM**_10_ | 2 | 3 | 12 | 1 |
| **PM**_2.5_ | 1 | 4 | 14 | 1 |
| **UFP** | 0 | 2 | 127 | 41 |

**Supplementary Table S5.** Number of mass spectrometry features significantly associated with each air pollutant and the interaction term with physical activity, after Bonferroni correction for multiple testing, in the TAPAS II study.

| **Air pollutant** | **N associations**^a^ | |
| --- | --- | --- |
|  | **Air pollution concentration** | **Interaction with**  **physical activity** |
| ***PM*_2.5_** | 5 | 0 |
| ***PM*_10_** | 6 | 0 |
| ***PM*_coarse_** | 11 | 0 |
| ***NO*_x_** | 3 | 0 |

^a^ Statistical models are adjusted for age, sex and BMI

**Supplementary Table S6.** Number of mass spectrometry features significantly associated with each air pollutant among healthy individuals, individuals with COPD or with IHD, after Bonferroni correction for multiple testing, in the Oxford Street II study.

| **Air pollutant** | **N associations**^a^ | | |  |
| --- | --- | --- | --- | --- |
|  | **Healthy** | **COPD** | **IHD** | **COPD and IHD** |
| ***CBLK*** | 38 | 98 | - | 41 |
| ***NO*_2_** | 142 | 117 | - | 26 |
| ***PM*_2.5_** | 129 | 85 | 409 | 2 |
| ***PM*_10_** | 156 | 68 | 443 | 0 |
| ***UFP*** | 51 | 50 | - | 7 |

^a^ Statistical models are adjusted for age, sex, BMI and caffeine intake. Annual exposure was not included in the models because of convergence issues.

*S6. MS/MS spectra of the identified compounds*

Phenylalanine ; m/z=166.0868 Da ; rt=2.04 min (Table 4)

Standard

Sample

Acyl Carnitine (C6:0-OH)  (1); m/z=276.1805 Da ; rt=2.22 min (Table 4)

Acyl Carnitine (C6:0-OH)  (2); m/z=276.1805 Da ; rt=2.40 min (Table 4)

Acyl Carnitine (Phenyl-acetyl carnitine); m/z=280.1543 Da ; rt=2.76 min (Table 4)

Caffeine ; m/z=195.0877 Da ; rt=3.19 min (Table 4)

Standard

Sample

Acyl Carnitine (10:3) (1) ; m/z=310.2013Da ; rt=4.17 min (Table 4)

Acyl Carnitine (10:3) (2) ; m/z=310.2013Da ; rt=4.35 min (Table 4)

Acyl Carnitine (11:1) ; m/z=328.2482 Da ; rt=5.12 min (Table 4)

MG (24:0) ; m/z=443.4101 ; rt=8.54 min (Table 4)

L-Tyrosine ; m/z=182.0812; rt=1.34 min (Table 5)

Standard

Sample

PC(16:0); m/z=502.3284 Da; rt=7.14 min (Table 5)

m/z=502.3284 Da corresponds to the [M+Na]^+^ adduct

PC (20:2) ; m/z=570.3553; rt=7.16 min (Table 5)

m/z=570.3553 Da corresponds to the [M+Na]^+^ adduct
